# Resveratrol Improves Heart Function by Moderating Inflammatory Processes in Patients with Systolic Heart Failure

**DOI:** 10.3390/antiox9111108

**Published:** 2020-11-11

**Authors:** Roland Gal, Laszlo Deres, Orsolya Horvath, Krisztian Eros, Barbara Sandor, Peter Urban, Szilvia Soos, Zsolt Marton, Balazs Sumegi, Kalman Toth, Tamas Habon, Robert Halmosi

**Affiliations:** 1 Division of Cardiology, 1st Department of Medicine, Medical School, University of Pecs, 7602 Pecs, Hungary; gal.roland@pte.hu (R.G.); deres.laszlo@pte.hu (L.D.); horvath.orsolya2@pte.hu (O.H.); sandor.barbara@pte.hu (B.S.); marton.zsolt@pte.hu (Z.M.); toth.kalman@pte.hu (K.T.); habon.tamas@pte.hu (T.H.); 2 Szentágothai Research Centre, University of Pecs, 7602 Pecs, Hungary; krisztian.eros@aok.pte.hu (K.E.); urban.peter@pte.hu (P.U.); balazs.sumegi@aok.pte.hu (B.S.); 3 HAS-UP Nuclear-Mitochondrial Interactions Research Group, 1007 Budapest, Hungary; 4 Department of Biochemistry and Medical Chemistry, Medical School, University of Pecs, 7602 Pecs, Hungary; 5 Division of Pulmonology, 1st Department of Medicine, Medical School, University of Pecs, 7602 Pecs, Hungary; soos.szilvia@pte.hu

**Keywords:** resveratrol, oxidative stress, inflammation, echocardiography, heart failure

## Abstract

The effects of resveratrol (RES) in heart failure have already been evaluated in animal models; however, in human clinical trials, they have not been confirmed yet. The aim of this study was to assess the effects of resveratrol treatment in systolic heart failure patients (heart failure with reduced ejection fraction or HFrEF). In this human clinical trial, 60 outpatients with NYHA (New York Heart Association) class II-III HFrEF were enrolled and randomized into two groups: receiving either 100-mg resveratrol daily or placebo for three months. At the beginning and at the end of the study echocardiography, a six-minute walk test, spirometry, quality of life questionnaire, lab test and RNA profile analysis were performed. The systolic and diastolic left ventricular function, as well as the global longitudinal strain, were improved significantly in the resveratrol-treated group (RES). Exercise capacity, ventilation parameters and quality of life also improved significantly in the RES group. In parallel, the cardiac biomarker levels (N-terminal prohormone of brain natriuretic peptide (NT-proBNP) and galectin-3) decreased in the treated group. The level of inflammatory cytokines decreased significantly after RES supplementation, as a consequence of the decreased expression level of leucocyte electron transport chain proteins. The main findings of our trial are that RES treatment added to the standard heart failure therapy improved heart function and the clinical condition by moderating the inflammatory processes in patients with HFrEF.

## 1. Introduction

Heart failure (HF) is a complex multifactorial syndrome caused by structural and/or functional cardiac abnormalities, resulting in a reduced cardiac output or elevated intracardiac pressures [1]. Heart failure prevalence is continuously rising throughout the world. In developed countries, approximately 2% of the adult population has heart failure.

Therapeutic developments over the past several decades have focused on blocking the neurohumoral (RAAS and adrenergic system) activation and on the inhibition of the breakdown of vasoactive peptides. Although this approach improved the survival of HFrEF (heart failure with reduced ejection fraction) patients [2], the outcome of the disease still remained poor [3,4,5,6,7,8]. Therefore, there is an overwhelming need for new therapies in heart failure. Novel drugs in experimental and clinical trials targeting myocardial contractility (e.g., omecamtiv mecarbil), cytokines (e.g., anti-TNFα), myocardial metabolism (e.g., perhexiline) or oxidative stress [9,10] are promising and may present an alternative approach in the treatment of heart failure in the future.

Oxidative stress also has an important role in the pathogenesis of different cardiac pathologies, such as ischemia-reperfusion injury, cardiac remodeling and heart failure [11,12]. Resveratrol (3,5,4-trihydroxystilbene), a natural phytoalexin found in a wide variety of plants (e.g., nuts, berries and grapes) is produced in response to environmental stress [13]. Given that resveratrol has a marked scavenger effect, it is not surprising that it can protect cells against oxidative damages [14,15,16,17]. Beyond its scavenger effect, resveratrol also has antioxidant effects in other ways. Resveratrol (RES) via the activation of the NAD^+^-dependent histone/protein deacetylase SIRT1 (silent information regulator) and AMPK (AMP-activated protein kinase) facilitates mitochondrial biogenesis in cardiomyocytes. This is accompanied by enhanced oxidative phosphorylation and a higher amount of high energy phosphates (CrP and ATP) that can result in increased contractility and efficient protection against oxidative stress [18,19,20]. Endothelial and vascular function can also be improved more by the RES treatment via decreasing the cholesterol and triglyceride levels via increasing the endothelial nitric oxide synthase (eNOS) activity and nitrogen oxide (NO) level, as well as due to its anti-inflammatory effects [21,22,23,24].

In systolic heart failure (HFrEF), the contractility of the heart muscle cells is decreased; moreover, a chronic low-intensity inflammation can be seen that can further aggravate heart failure. Our workgroup demonstrated previously that resveratrol supplementation in a post-infarct rodent model prevented the development of heart failure [25]. RES improved the left ventricular function, as well as decreased myocardial fibrosis, oxidative stress and the amount of proinflammatory proteins (cyclooxygenase-2 (COX-2) and iNOS). Other workgroups showed similar results in various animal heart failure models. However, in human clinical trials, the effect of RES has not yet been confirmed [13,26].

The aim of our study was, therefore, to assess the effects of resveratrol treatment on the left ventricular function and on the exercise capacity, as well as on the biomarker and inflammatory cytokine levels in HFrEF.

## 2. Materials and Methods

### 2.1. Study Design

**Figure 1:**
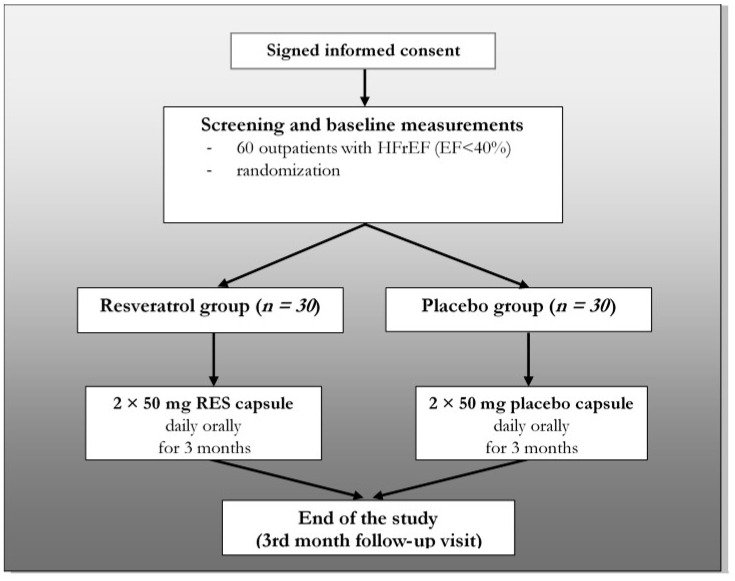
Study design. HFrEF: heart failure with reduced ejection fraction, N: number of patients and RES: resveratrol.

Our trial was a single-center, double-blind, randomized, placebo-controlled study. The study design is summarized in Figure 1. The study was conducted in accordance with the principles stated in the Declaration of Helsinki (1996), International Conference on Harmonization Good Clinical Practice, as well as local and national regulations. The protocol of the trial was approved by the Regional Ethics Committee of the University of Pecs (license number: 5830). All patients provided written informed consent prior to any study-related procedures.

### 2.2. Eligibility

The main inclusion and exclusion criteria are listed in Table 1. Briefly, 60 stable outpatients (ages: 66.7 ± 2.01 years, 17 women and 43 men) with HFrEF in NYHA (New York Heart Association) class II or III were enrolled between 01 March 2016 and 30 November 2017 into our study (ejection fraction (EF) <40% and ischemic/non-ischemic origin: 34/26). They were randomized into two groups (resveratrol group and placebo group). one-hundred milligrams of resveratrol was administered orally (2 × 50 mg) for 3 months in the RES group (*n* = 30) and placebo capsule in the other group (*n* = 30).

The resveratrol and the matching placebo capsules were purchased from Argina Nutraceuticals Ltd. (Fót, Hungary). The resveratrol capsule is commercially available and has an official license for being marketed.

All the involved patients were in the evidence-based drug treatment of HFrEF, including angiotensin-converting enzyme (ACE) inhibitors (or ARBs—angiotensin receptor blockers), beta-blockers, mineralocorticoid receptor antagonists and ivabradine. None of the patients received ARNI (angiotensin receptor-neprilysin inhibitor). The preventive drug regime and the used doses were based on the actual European Society of Cardiology (ESC) heart failure guideline [1]. The patients had baseline visits and follow-up visits after 1 month and after 3 months of treatment. During visits, the compliance of patients was checked, and at the final visit (3-month follow-up visit), they were asked to return the remained capsules. During the whole study period, subjects were in stable clinical conditions and received unchanged medical therapy (Table 2). The baseline characteristics of the randomized patients are detailed in Table 2. There were no significant differences in epidemiological characteristics between the placebo and RES-treated groups at the baseline.

On the day of randomization and 3-months-later physical examination, blood pressure and weight measurement, echocardiography, lab test, six-minute walk test (6MWT), spirometry and quality of life questionnaire (QoL test) were performed. At the 1-month follow-up visit, only a physical examination, blood pressure, weight measurement and lab test were done (Table 3).

### 2.3. Statistical Analysis

SPSS statistical software, version 25 (IBM Corp. Released 2017. IBM SPSS Statistics for Windows, Version 25.0. Armonk, NY: IBM Corp.) was used to conduct a descriptive analysis and to describe the sample. After using the Kolmogorov-Smirnov test to check the normality of the data distribution, differences of the mean values within the groups were analyzed by repeated-measures ANOVA with a Greenhouse–Geisser correction. Differences between the groups were calculated by one-way ANOVA test. Data are expressed as mean ± S.E.M. Significance level was defined as *p* < 0.05. The homogeneity of the groups was tested by Levene’s F-test. The nonparametric Friedman test (post-hoc analysis with Wilcoxon signed-rank test) was applied to analyze the potential changes of the quality of life of the patients.

### 2.4. Blood Pressure and Body Weight Measurement

Body weight and blood pressure were measured during randomization at 1-month and at 3-month follow-up visits. For body weight measurements, a high-quality calibrated digital scale was used (CAS PB-150 digital scale; CAS Corporation, Tustin, CA, USA). Blood pressures were measured using an automated electronic device after a 5-min resting; the location of the cuff was the upper arm at the level of the heart (OMRON M2 Intellisense, Tokyo, Japan). The technique of the measurement was based on the actual hypertension guideline [27].

### 2.5. Laboratory Test

Laboratory analysis performed on blood samples that were extracted from the cubital vein after a 12-h-long fasting period. From blood samples, NT-proBNP; fasting lipid levels (total cholesterol, low-density lipoprotein (LDL)—cholesterol, high-density lipoprotein (HDL) -cholesterol and triglycerides); troponin T; glycosylated hemoglobin-HgbA1c; serum albumin; renal and liver function; serum iron parameters and quantitative and qualitative blood cell counts were measured in the Department of Laboratory Medicine, University of Pecs, Pecs, Hungary.

Special parameters (galectin-3, interleukin (IL)-1 and IL-6) were determined using an ELISA method in the Szentagothai Research Centre, University of Pecs. Blood samples were collected into Vacutainer tubes containing EDTA and protease inhibitor. Galactin-3 level (Invitrogen™ Human Galectin-3 ELISA Kit, Thermo Fisher Scientific, EHLGALS3, Waltham, MA, USA), IL-1 cytokine level (Invitrogen™ Human IL-1 R4 (IL1RL1) ELISA Kit, Thermo Fisher Scientific, EHIL1RL1, Waltham, MA, USA) and IL-6 (Invitrogen™ Novex IL-6 Human ELISA Kit, Thermo Fisher Scientific, KHC00612.3.3., Waltham, MA, USA) cytokine levels were determined by the enzyme immunoassay method.

### 2.6. Echocardiography

Transthoracic echocardiography was performed for noninvasive evaluation of cardiac structure and function at baseline and after the 3-month-long treatment. We used a GE Vivid E9 ultrasound imaging device (GE Healthcare, Chicago, IL, USA). Structures were visualized from parasternal long axis; short axis and apical four, two and five-chamber views. M-mode (one-dimensional mode), 2D-mode, 3D-mode, PW (pulsed-wave), CW (continuous wave) and tissue Doppler mode imaging were used to determine the left ventricular ejection fraction (EF, %), the diameters (mm) and volumes (mL) of the left ventricle and left atrium, the diastolic function (E/A and E/E’), the global longitudinal strain of the left ventricle (GLS, %), the wall thicknesses of the left ventricle (mm) and the right ventricular parameters. The EF was measured by two different methods (“Simpson” and “Quinone”). The investigators were blinded to the protocol; thus, they did not know whether the patient was taking resveratrol or placebo.

### 2.7. Six-Minute Walk Test (6MWT)

The 6-min walk test (6MWT) is a submaximal exercise test that measures the walking distance for 6 min. The test was performed on a 30-m-long section of the corridor in our department according to the guidelines of the American Thoracic Society (ATS). The patients were at rest comfortably for 10 min prior to the test. The supervisor was a study nurse, who was blinded to the protocol. After 6 min or if the patient could not go any further, the test was stopped, and the distance, as well as the reason for stopping (dyspnea, fatigue, chest pain, etc.), were recorded [28].

### 2.8. Spirometry

Spirometry is a routine method to evaluate the lung function, and it has many uses, including chest surgery or the grading of chronic obstructive pulmonary disease (COPD), etc. It is well-known that heart failure can influence the pulmonary function, which may include combined obstructive (e.g., significant FEV1 reduction) and restrictive changes [29].

We used a PISTON PDD 301/s spirometer (Piston Ltd., Budapest, Hungary) for measuring the basic resting lung parameters of the patients at baseline and 3 months later.

During the test, the patient wore a soft nose clip to prevent air escaping through the nose, as well as a mouthpiece filter to prevent the spread of microorganisms. During the test, patients were asked to take the deepest breath they could and then exhale into the sensor as hard and as long as possible. Generally, a period of quiet breathing in and out from the sensor precedes the test, and the rapid breathing in (forced inspiratory part) comes before the forced exhalation. We determined the most common parameters in the percentage of the reference values, such as forced vital capacity (FVC); forced expiratory volume (FEV_1_); FEV_1_/FVC ratio; peak expiratory flow (PEF); forced expiratory flow 25–75% (FEF 25–75); maximal expiratory flows (MEF 25%, MEF 50% and MEF 75%) and inspiratory vital capacity (IVC).

### 2.9. Quality of Life Questionnaire (QoL Test)

Quality of life (QoL) in heart failure patients is severely compromised by the symptoms of the disease. Quality of life is also a good predictor of mortality and the need for hospitalization. For the assessment of QoL, we used the “Euro QoL five-dimension” (EQ-5D) questionnaire. Among health-related quality of life (HR QoL) questionnaires, EQ-5D has gained widespread use due to its simplicity to administer, score and interpret. This scoring system consists of five dimensions (mobility, self-care, usual activities, pain/discomfort and anxiety/depression), each with three levels of response or severity (no problems—2 point, some problems—1 point or extreme problems—0 point). In addition to the index-based scoring system, the visual analog scale (VAS) component of the EQ-5D enables the patient to place their current health state on a range from 0 (worst imaginable health state) to 100 (best imaginable health state) [30].

### 2.10. RNA Isolation, RNA-Seq Library Preparation and Sequencing

QIAamp RNA Blood Mini Kit was used for purification of total RNA from the whole blood. During the QIAamp procedure for purification of RNA from blood, erythrocytes are selectively lysed, and leukocytes are recovered by centrifugation. The leukocytes are then lysed using highly denaturing conditions that immediately inactivate RNases, allowing the isolation of intact RNA. After homogenization of the lysate by a brief centrifugation through a QIA shredder spin column, ethanol is added to adjust the binding conditions, and the sample is applied to the QIAamp spin column. RNA is bound to the silica membrane during a brief centrifugation step. Contaminants are washed out, and total RNA is eluted in 30 μL or more of RNase-free water.

RNA concentrations were measured using Qubit 3.0 (Invitrogen, Carlsbad, CA, USA). The RNA quality was verified on an Agilent 2100 Bioanalyzer using an RNA 6000 Nano Kit (Agilent Technologies, Santa Clara, CA, USA). High-quality (RIN > 8) RNA samples were processed for library preparation. Further sample preparation was conducted by Illumina TruSeq^®^ Stranded Total RNA sample preparation guide.

### 2.11. RNA-Sequencing Data Processing and Analysis

Paired-end fastq files were imported into the CLC Genomics Workbench (CLC Bio, version 12, Hilden, Germany). Adaptor sequences and bases with low quality were trimmed. Reads containing more than 2 ambiguous bases were discarded. Preprocessed sequencing reads were aligned to the gene regions and transcripts of the GRCh38 human genome.

Differential expression of genes at the end of the study was assessed in each experimental group compared to baseline expression values while controlling cross-checking of the patient IDs of paired data. Differentially expressed genes (DEGs) were filtered out, using a threshold of fold change (FC) absolute value > 1.5 and false discovery rate (FDR)-adjusted *p*-value < 0.05. DEGs were visualized on a heat map using CLC Genomics Workbench 12. Hierarchical clustering was conducted, using Euclidean distances and complete linkages.

## 3. Results

### 3.1. The Effect of Resveratrol on Laboratory Parameters

Laboratory parameters did not show significant changes in either group after one month of treatment compared to the baseline values (data not shown). There was no significant change in renal function, liver function and quantitative and quality blood counts in either group after the three-month follow-up period compared to the baseline values. Cardiac troponin T, iron parameters and glycosylated hemoglobin (HgbA1c) were also not influenced significantly by the resveratrol treatment during the study period. The total cholesterol (4.74 ± 0.22 mmol/L vs. 4.52 ± 0.24 mmol/L, *p* < 0.05) and LDL-cholesterol (3.09 ± 0.23 mmol/L vs. 2.79 ± 0.23 mmol/L, *p* < 0.05) levels were significantly lower in the RES group by the end of the follow-up period; however, in the placebo group, no significant alterations could be seen compared to the baseline values (Table 4). Unfortunately, the HDL-cholesterol level was also slightly but significantly lower in the RES group (1.33 ± 0.06 mmol/L vs. 1.24 ± 0.07 mmol/L, *p* < 0.05 vs. RES-Baseline) after three months.

### 3.2. Inflammatory Parameters

**Figure 2:**
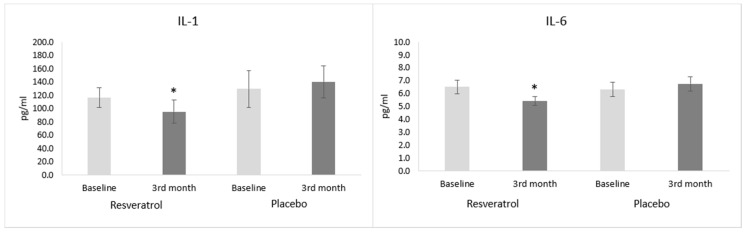
Effect of resveratrol on inflammatory cytokines in heart failure patients. Values are expressed as mean ± SEM. * *p* < 0.05 vs. placebo group at the 3rd month. Baseline: measured values at randomization in resveratrol or in placebo group and 3rd month: patients treated with resveratrol or placebo for 3 months. IL: interleukin.

The acute-phase proteins (CRP and ferritin) and white blood cell count did not show significant changes in either group during the three-month-long follow-up period (Table 4). The levels of IL-1 (95.61 ± 17.74 pg/mL vs. 140.65 ± 24.04 pg/mL, *p* < 0.05) and IL-6 (5.42 ± 0.35 pg/mL vs. 6.76 ± 0.56 pg/mL, *p* < 0.05) measured by ELISA, however, were significantly lower in the RES-treated group at the end of the study period compared to the placebo group (Figure 2).

### 3.3. Biomarkers of Heart Failure

**Figure 3:**
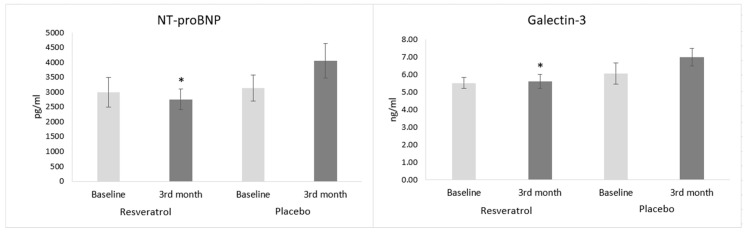
Effects of resveratrol on the biomarkers of heart failure. Values are expressed as mean ± SEM. * *p*< 0.05 vs. placebo group at the 3rd month. Baseline: measured values at randomization in the resveratrol or in the placebo group and 3rd month: patients treated with resveratrol or placebo for 3 months. NT-proBNP: N-terminal prohormone of brain natriuretic.

There was no significant difference in the NT-proBNP level between the RES and placebo groups at the baseline (2998 ± 507 pg/mL vs. 3139 ± 446 pg/mL, NS); however, by the end of the treatment period, the NT-proBNP level was significantly lower in the RES group than in the placebo group (2760 ± 346 pg/mL vs. 4054 ± 577 pg/mL, *p* < 0.05). This change suggests that RES inhibited the elevation of the plasma NT-proBNP level, a parameter which showed the severity of heart failure. In parallel with NT-proBNP, the galectin-3 level also showed a significant difference between RES and the placebo group at the end of the study (5.61 ± 0.42 ng/mL vs. 6.98 ± 0.54 ng/mL, *p* < 0.05) (Figure 3).

### 3.4. BMI, General Hemodynamic and ECG Parameters

Body mass index (BMI) alterations were not significant in either the resveratrol (29.31 ± 0.99 kg/m^2^ vs. 29.06 ± 1.01 kg/m^2^, NS) or in the placebo group (31.04 ± 1.46 kg/m^2^ vs. 31.34 ± 1.39 kg/m^2^, NS) after three months compared to the baseline (Table 5). Similarly, no significant alterations could be seen in the blood pressure, in the heart rate and in the ECG parameters after one month (data not shown) and at the end of the study (Table 5).

### 3.5. The Effect of Resveratrol on Echocardiographic Parameters

**Figure 4:**
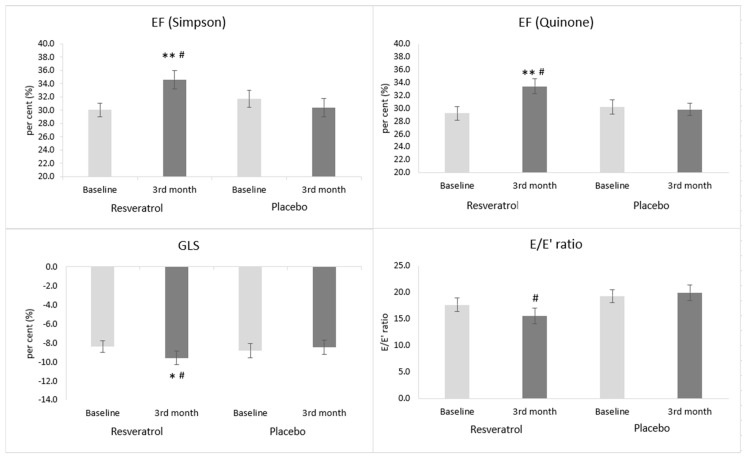
The effect of resveratrol on the left ventricular systolic and diastolic functions. Values are expressed as mean ± SEM. * *p* < 0.05, 3rd month values of the resveratrol group compared to the baseline values. ** *p* < 0.01, 3rd month values of the resveratrol group compared to the baseline values. # *p* < 0.05, resveratrol vs. placebo group at the 3rd month. Baseline: measured values at randomization in the resveratrol or in the placebo group; 3rd month: patients treated with resveratrol or placebo for 3 months. E: early diastolic ventricular filling velocity, E’: early diastolic mitral annular velocity, EF: ejection fraction and GLS: global longitudinal strain of the left ventricle.

The echocardiographic parameters of the patients did not differ significantly between the groups at the beginning of the study. The left ventricular EF measured by two different methods was improved significantly due to resveratrol administration by the end of the treatment period compared to the baseline (Quinone method: 29.19 ± 1.04% vs. 33.40 ± 1.20%, *p* < 0.01 and Simpson method: 30.06 ± 1.04% vs. 34.60 ± 1.44%, *p* < 0.01) (Table 6 and Figure 4). In parallel with this result, the left ventricular stroke volume (SV) also increased, and the left ventricular end-systolic volume (LVESV) decreased significantly in the RES group. Global longitudinal strain (GLS,%) of the LV showed a significant improvement (−8.40 ± 0.62 % vs. −9.58 ± 0.73%, *p* < 0.05) in the treated group by the end of the follow-up compared to the baseline. In contrast, in the placebo group, no significant change could be observed.

In the case of LV diastolic function, a significant difference in the E/A (1.18 ± 0.15 vs. 1.54 ± 0.19, *p* < 0.05) and E/E’ ratios (15.55 ± 1.43 vs. 19.94 ± 1.43, *p* < 0.05) was observed between the resveratrol and placebo groups after the three-month-long treatment.

In addition, the LAESV, LA long axis, RVIDd, RVIDs and RAEDV were also decreased significantly in the RES group compared to the RES baseline values, as well as LAEDV, RAESV, IVC and TAPSE, which also showed significant differences between the resveratrol and placebo groups after three months (Table 7).

### 3.6. The Effect of Resveratrol on Exercise Capacity

The six-minute walk distance increased significantly in the resveratrol group (from 275 ± 19 m to 298 ± 22 m, *p* < 0.05) by the end of the treatment period compared to the baseline; however, in the placebo group, there was no apparent change (NS).

### 3.7. The Effect of Resveratrol on the Respiratory Parameters

The treatment with resveratrol resulted in significantly improved FVC (75.38 ± 3.31% vs. 79.27 ± 3.07%, *p* < 0.05) by the end of the study period. Other expiratory parameters did not show any changes compared to the baseline values (Table 8). However, the inspiratory vital capacity (IVC) also increased significantly by resveratrol administration (83.85 ± 3.65% vs. 90.27 ± 3.39%, *p* < 0.05) compared to the baseline.

### 3.8. The Effect of Resveratrol on the Quality of Life in Patients with Systolic Heart Failure

For the assessment of QoL the “Euro QoL five-dimension” (EQ-5D) questionnaire was used. The main domains of the test did not differ significantly among the groups at the beginning of the study. Our nonparametric statistical analysis showed significant improvement in the mobility (Z = −2.236, *p* < 0.05), usual activities (Z = −2.646, *p* < 0.05) and anxiety/depression (Z = −2.236, *p* < 0.05) but did not reveal any significant difference in self-care and pain/discomfort of the patients in their resveratrol-treated group after three months compared to the baseline values. Summary, RES administration resulted in a significantly improved percentage of the subjective health state (54.86 ± 2.78% vs. 61.17 ± 2.72%; Z = −3.146, *p* = 0.002), but there was no significant change in the placebo group (49.43 ± 3.15% vs. 48.17 ± 3.34%; Z = −0.631, NS).

### 3.9. Sequence Analysis and Differential Expression

**Figure 5:**
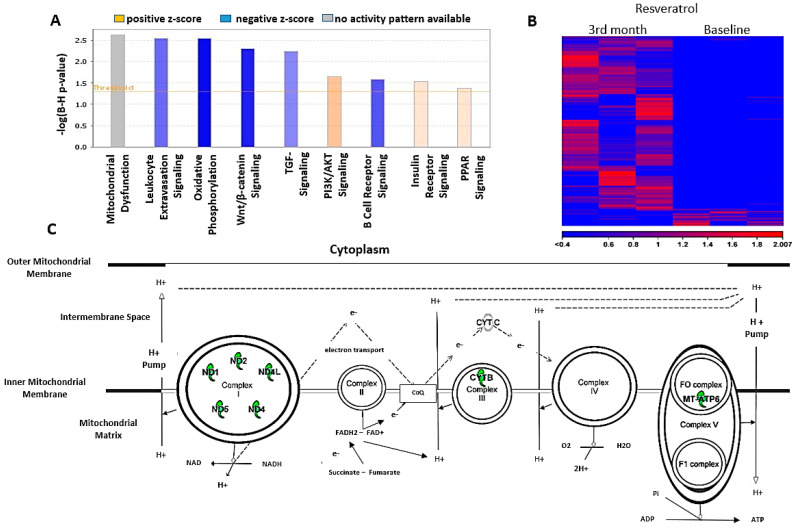
(**A**) Summary of the canonical pathways impacted by the differentially expressed genes (DEGs) due to the resveratrol treatment (*n* = 7). Coloring of the bars represents the predicted activation state of the given pathway based on Z-score calculations applied on the differential expression of the genes in our study, included in the given canonical pathway. (**B**) Resveratrol supplementation induced expressional alterations in human leukocytes. Heat map visualizing the expression values of the differentially expressed genes (the most demonstrative 3 patients) significant in the resveratrol-treated patients. Hierarchical clustering was conducted on Euclidean distances and complete linkages. (**C**) The effect of RES on electron transport chains (ETC) in leukocytes. The canonical pathway of oxidative phosphorylation (generated by the IPA /Ingenuity Pathway Analysis/ program) depicting (green) only the members of ETC complexes demonstrating suppressed expression due to the resveratrol treatment. ADP: adenosine diphosphate, ATP: adenosine triphosphate, CoQ: coenzyme Q, MT-ATP6: gene of mitochondrial ATP synthase, NAD: nicotinamide adenine dinucleotide, ND: NADH dehydrogenase and CYT: cytochrome.

At the end of the study, the mRNA analysis of seven patients showed that, in the resveratrol group, 222 genes were differently expressed ((FC (fold change) abs. value >1.5 and FDR *p*-value < 0.05)) compared to the baseline values (Figure 5A,B).

The main finding in this trial was that the resveratrol treatment suppressed the expression of seven genes of mitochondrial ETC (electron transport chain) members in leukocytes, including MT-ATP6 (ATP synthase F0 subunit 6—complex V), MT-CYB (cytochrome b), MT-ND1 (NADH dehydrogenase, subunit 1—complex I), MT-ND2 (NADH dehydrogenase, subunit 2—complex I), MT-ND4 (NADH dehydrogenase, subunit 4—complex I), MT-ND5 (NADH dehydrogenase, subunit 5—complex I) and MT-ND4L (NADH dehydrogenase, subunit 4L—complex I) (Figure 5C). However, the genes of complex II and complex IV were not affected by resveratrol. Our results revealed that resveratrol had an inhibitory effect on ATP synthesis via the oxidative phosphorylation; however it did not lead to mitochondrial dysfunction in leukocytes, because it has no negative effect on the proteins affecting the mitochondrial quality control (Figure 5A).

## 4. Discussion

Our present work firstly proved that resveratrol beneficially influences heart failure in a randomized double-blind clinical trial (RCT). The major findings of our trial are that the resveratrol treatment improved heart function, exercise tolerance, several spirometry parameters and quality of life and decreased the level of cholesterol and inflammatory cytokines in systolic heart failure patients.

It is well-established that resveratrol has various beneficial effects on the cardiovascular system. It has a marked antioxidant effect due to its scavenger capability and due to enhancing the antioxidant enzyme production (SOD, CAT and eNOS), as well as decreasing the amount of prooxidant enzymes (NOXs and MPO) [31,32,33]. Therefore, resveratrol also has a marked anti-inflammatory and antiplatelet effect [34,35,36]. Resveratrol also has beneficial effect on metabolic parameters; it can decrease the cholesterol level and improve the insulin resistance [37,38]. However, these data are derived almost completely from preclinical studies.

It was published previously by our workgroup that resveratrol in a murine post-infarct heart failure model improves the heart function and decreases myocardial fibrotic remodeling via its anti-inflammatory effect and via blocking the profibrotic intracellular signaling routes [25]. Other workgroups showed similar results in various animal heart failure models; however, in a human clinical trial, the effect of RES has not been confirmed yet [13,26].

In this one-center double-blind RCT, the baseline characteristics of symptomatic systolic heart failure (HFrEF) patients were well-balanced due to the used randomization method (adaptive minimization) [39]. Guideline-directed medical treatment (GDMT) was administered in the maximal tolerated dose, and almost every patient was given ACEI or ARB and beta-blockers, and only one-quarter of the patients was not on MRA treatments (Table 2).

In our heart failure patients, resveratrol supplementation improved the systolic left ventricular function expressed as the ejection fraction (EF). The EF was measured in two different ways, by using the Quinones and the Simpson methods. We chose the Quinones method instead of the Teichholz’s method, because according to the literature, it is better correlation with volumetric methods [40]. Nowadays, however, the volumetric Simpson method is by far the most commonly used method for quantifying left ventricular function. In the case of both methods, a similar increase could be seen in the resveratrol-treated group; however, in the placebo group, no change was observable.

Global longitudinal strain (GLS) is another measure of LV global function that also correlates with the extent of myocardial fibrosis in patients with HFrEF [41]. In the placebo-treated group, the GLS value remained unchanged by the end of the treatment period. The resveratrol treatment, however, caused an improvement of the GLS value in parallel with other measures indicating systolic heart function. Moreover, GLS can show the extent of myocardial remodeling, especially myocardial fibrosis. In parallel with the improvement of systolic heart function, diastolic heart function also got better (i.e., the E/e’ ratio decreased) in the resveratrol group. In the placebo group the diastolic function was unchanged.

Natural polyphenols could improve heart function in a wide variety of experimental heart failure models [42,43,44]. However, in human clinical trials, there are only a few data regarding the effect of resveratrol treatment on heart function. Our workgroup proved previously that a low-dose resveratrol treatment (10 mg/day) improved the diastolic function in patients with coronary artery disease (CAD), while, in the case of systolic LV function, only a favorable tendency was present (NS) [45]. In another small trial also conducted in CAD patients using a higher dose (100 mg/day) of resveratrol, both the systolic and diastolic function improved.

Not only the heart function but, also, the exercise tolerance determined by the 6MWT showed significant improvement in resveratrol-treated patients by the end of the three-month-long treatment period. This result is in accordance with the preclinical results carried out in several murine models [46]. However, there are no clinical data regarding the effect of resveratrol on the physical exercise capacity in heart failure patients yet. In an interesting trial, Voduc N. and his coworkers proved that resveratrol itself in healthy people has no positive effect on exercise capacity and on the maximal oxygen consumption (VO_2_max) [47]; moreover, Glieman L. et al. demonstrated that, in aged men, the resveratrol treatment blunted the positive cardiovascular effects of exercise and moderated the increase of VO_2_max caused by the training program [48]. Therefore, the increase of exercise capacity can be predominantly the consequence of the improvement of heart function in heart failure patients.

The analysis of cardiac biomarkers strengthens the findings regarding heart function and exercise tolerance. Galectin-3, which is secreted by macrophages, has been known for its significant role in mediating cardiac fibrosis and inflammation [49]. These data were also proved in a human clinical examination [50]. In our trial, the resveratrol treatment decreased the galectin-3 level, but in the placebo group, no change could be seen. This result supports the beneficial changes that occurred in the case of GLS, because GLS is directly proportional to the extent of interstitial fibrosis [41].

NT-proBNP is the most widely used biomarker in the diagnosis and risk stratification of heart failure. It shows a strong correlation with the severity of heart failure, although its value can be variable due to alterations in the volume status of patients. In our trial, the resveratrol supplementation decreased the level of NT-proBNP (NS), and in the placebo group, a worsening tendency could be seen, so by the end of the treatment period, a significantly lower natriuretic peptide level was achieved in the RES group than in the placebo group. In a study conducted in stable CAD patients, Militaru and coworkers found that resveratrol decreases the NT-proBNP level even without overt heart failure [51]. The continuously increasing level of heart failure biomarkers (NT-proBNP and galectin-3) in the placebo group can be a consequence of the progression of the disease. According to the literature, there was a linear relationship between NT-proBNP and the number of hospitalization, as well as mortality, due to heart failure [52].

The improvement of left ventricular heart function in the resveratrol-treated group is in-line with the decreased volume retention and with the decongestion of organs. It is known that extravascular fluid accumulation in the lungs is accompanied by the alteration of several spirometry measures, e.g., forced vital capacity (FVC) and inspiratory vital capacity (IVC) [53,54]. Moreover, Gehlback et al. showed that, after heart transplantation, not only the ventilation volumes but the airflow velocity (FEV1) was also better [54]. Due to resveratrol supplementation in the present trial, FVC and IVC increased significantly by the end of the treatment period. However, in the case of airway obstruction parameters, no significant changes could be seen in this study. FEV1 showed only a mild, nonsignificant improvement in the RES group.

The level of inflammatory cytokines, IL-1 and IL-6 decreased in the resveratrol-treated heart failure patients compared to the placebo group. Similar results were seen in the literature [55]. This favorable change is parallel with the level of galectin-3β that mediates inflammation. The levels of inflammatory cytokines produced by the activated leukocytes are inversely proportional to the systolic left ventricular function [56]. The increasing level of IL-1 and IL-6 in the placebo group can be explained by the slow progression of heart failure in our enrolled population. The correlation between inflammation and adverse cardiovascular outcomes in heart failure was documented. Essentially, heart failure progression was attributed to sustained proinflammatory cytokine signaling, based on the observation that proinflammatory cytokines were elevated and continuously worsened during the progression of the disease [57]. Interestingly, the mRNA profile analysis of leukocytes revealed a significant decrease in genes encoding several mitochondrial respiratory proteins. Resveratrol, however, did not interfere with the expression of various proteins playing a part in the mitochondrial quality control. Moderating the production of ETC proteins in leukocytes can decrease the oxidative phosphorylation in leukocytes, which is directly proportional to their activity. The decreased production of proinflammatory cytokines in this work can be a sign of the moderated activity of leukocytes. This anti-inflammatory effect can be an important aspect of the resveratrol treatment in heart failure patients, besides its well-known mitochondrial protective effects in cardiomyocytes.

## 5. Conclusions

In this human clinical trial, the positive effects of resveratrol were proved in systolic heart failure (HFrEF) patients added to the standard therapy. The resveratrol treatment improved several parameters of heart function, exercise tolerance and quality of life. Moreover, resveratrol exerted an anti-inflammatory effect measured by the decrease of levels of inflammatory cytokines (IL-1 and IL-6). According to our results, the decreased activity of leukocytes can be an important mechanism of resveratrol, and it can contribute to its cardioprotective effect in heart failure.

## 6. Limitations of the Study

This study has some potential limitations. Although the study was double-blinded and randomized, (1) the number of the enrolled patients was quite low (*n* = 30/group), and (2) the follow-up period was relatively short. Due to these limitations, the results, including RNA sequencing, have to be interpreted cautiously. In the future, adequately powered randomized, controlled trials reporting patient-relevant outcomes with long-term follow-up periods are required to properly prove the efficacy of resveratrol supplementation in systolic heart failure, as well as to understand the exact biochemical and cellular mechanism.

## Figures and Tables

**Table 1:** Inclusion and exclusion criteria.

Inclusion Criteria	Exclusion Criteria
Stable HF patient	Hospitalization in the last 3 months
LVEF < 40%	LVEF ≥ 40%
NYHA II or III class at screening	NYHA IV class
NT-proBNP level > 125 pg/mL	≤ 18 or ≥ 85 years of age
GDM treatment of HFrEF according to the current ESC guideline Subject has provided informed consent	Known sensitivity to any components of the capsules
	Cardiac surgery or intervention within 30 days prior to randomization
	Systolic blood pressure > 180 Hgmm or < 90 Hgmm, or diastolic blood pressure > 90 Hgmm
	Heart rate >110 beats per minute or <50 beats per minute
	eGFR < 20 mL/1.73 m^2^/min or hepatic impairment defined by ALT or AST ≥ 2× upper limit of normal (ULN) at baseline
	Participation in a concurrent trial
	Refusal to give informed consent

ALT: alanine aminotransferase, AST: aspartate aminotransferase, eGFR: estimated glomerular filtration rate, GDM: guideline-directed medication, HF: heart failure, HFrEF: heart failure with reduced ejection fraction, LVEF: left ventricle ejection fraction, NT-proBNP: N-terminal prohormone of brain natriuretic peptide, NYHA: New York Heart Association, ULN: upper limit of normal.

**Table 2:** Baseline characteristics of the study population.

Baseline Characteristics	Resveratrol (*n* = 30)	Placebo (*n* = 30)
Age (year)	65.87 ± 1.91	67.53 ± 2.14
Male	22 (73%)	21 (70%)
Ejection fraction (%)	30.06 ± 1.04	31.70 ± 1.27
NT-proBNP (pg/mL)	2998 ± 507	3139 ± 446
Serum creatinine (μmol/L)	99.77 ± 4.42	104.47 ± 4.82
Systolic BP (Hgmm)	132.47 ± 3.41	128.77 ± 3.80
Diastolic BP (Hgmm)	79.10 ± 2.33	80.13 ± 2.66
Heart rate (beat/min)	72.20 ± 2.75	76.93 ± 2.50
Etiological factors		
Ischemic heart disease	17 (56.7%)	17 (56.7%)
Non-ischemic (alcohol, chemotherapy, myocarditis)	13 (43.3%)	13 (43.3%)
Risk factors, comorbidities		
Hypertension	22 (73%)	23 (76%)
Diabetes	13 (43%)	14 (46%)
Smoking	11 (36%)	8 (27%)
Pulmonary diseases (asthma, COPD)	7 (23%)	8 (27%)
BMI (kg/m^2^)	29.3 ± 0.9	30.4 ± 1.3
Target heart rate (<70/min)	23 (76.7%)	20 (66.7%)
Atrial fibrillation	7 (23%)	10 (33.3%)
Concomitant treatment		
ACE-inhibitor/ARB	28 (93%)	29 (97%)
Beta-blocker	29 (97%)	28 (93%)
MRA	23 (76.7%)	21 (70%)
Ivabradine	6 (20%)	6 (20%)
Diuretics		
Loop diuretics (furosemide, etacrynic acid)	27 (90%)	28 (93%)
Thiazide or thiazide-like diuretics (hypothiazide, indapamide, etc.)	8 (27%)	9 (30%)
Device therapy		
CRT-P/D	9 (30%)	7 (23.2%)
ICD	4 (13%)	3 (10%)

Values are expressed as mean ± SEM. There were no significant differences in patient characteristics between the RES (resveratrol) and placebo-treated groups at the baseline. ACEI: angiotensin-converting enzyme inhibitors, ARB: angiotensin receptor blocker, BMI: body mass index, BP: blood pressure, COPD: chronic obstructive pulmonary disease, CRT-P/D: cardiac resynchronization therapy-pacemaker/defibrillator, ICD: implantable cardioverter defibrillator, MRA: mineralocorticoid receptor antagonist and NT-proBNP: N-terminal prohormone of brain natriuretic peptide.

**Table 3:** Timetable of the study.

Examinations	Randomization	1-Month	3-Month
Physical examination	✓	✓	✓
Blood pressure monitoring	✓	✓	✓
12-lead ECG	✓	✓	✓
Lab test	✓	✓	✓
NT-proBNP level	✓		✓
mRNA analysis	✓		✓
Echocardiography	✓		✓
6MWT	✓		✓
Quality of life test (EQ-5D)	✓		✓
Spirometry	✓		✓

ECG: electrocardiography, EQ-5D: Euro quality of life-five dimension questionnaire, NT-proBNP: N-terminal prohormone of brain natriuretic peptide, mRNA: messenger ribonucleic acid and 6MWT: 6-min walk test.

**Table 4:** Effect of resveratrol treatment on the lab parameters.

Lab Parameters	Baseline		3rd Month	
	Resveratrol	Placebo	Resveratrol	Placebo
Hgb_A1c_ (%)	6.37 ± 0.18	6.70 ± 0.24	6.38 ± 0.20	6.67 ± 0.23
BUN (mmol/L)	7.75 ± 0.45	8.80 ± 0.84	8.26 ± 0.44	8.75 ± 0.93
Se creatinine (μmol/L)	99.77 ± 4.42	104.47 ± 4.82	102.19 ± 5.71	109.31 ± 5.92
TC (mmol/L)	4.74 ± 0.22 ^#^	4.15 ± 0.23	4.52 ± 0.24 * ^#^	4.04 ± 0.28
HDL-C (mmol/L)	1.33 ± 0.06	1.29 ± 0.08	1.27 ± 0.07 *	1.25 ± 0.06
LDL-C (mmol/L)	3.09 ± 0.23 ^#^	2.51 ± 0.21	2.79 ± 0.23 * ^#^	2.41 ± 0.23
TG (mmol/L)	1.91 ± 0.20 ^#^	1.61 ± 0.17	1.93 ± 0.21 ^#^	1.57 ± 0.22
ALT (IU/L)	25.87 ± 1.71	28.86 ± 1.58	27.33 ± 1.80	30.62 ± 2.39
AST (IU/L)	22.73 ± 2.35	28.07 ± 1.99	25.27 ± 3.42	26.93 ± 1.99
cTroponin T (ng/L)	23.26 ± 3.13	23.60 ± 2.56	22.25 ± 2.71	25.52 ± 2.91
Se albumin (g/L)	44.20 ± 0.56	44.82 ± 0.53	45.45 ± 0.58	46.05 ± 0.51
Se Fe (μmol/L)	15.11 ± 1.10	14.27 ± 1.13	15.54 ± 1.08	14.83 ± 1.02
Transf. sat. (%)	21.59 ± 0.84	20.08 ± 1.58	21.57 ± 1.60	20.37 ± 1.39
Se ferritin (μg/L)	151.1 ± 19.60	148.2 ± 18.46	138.5 ± 20.5	133.6 ± 15.85
CRP (mg/L)	4.66 ± 0.50	5.33 ± 0.83	4.23 ± 0.67	4.94 ± 0.73
WBC (G/L)	7.41 ± 0.33	7.73 ± 0.37	7.04 ± 0.29	7.78 ± 0.35
Hct (%)	42.00 ± 1.02	41.71 ± 0.90	41.69 ± 1.06	41.89 ± 0.94
PLT (G/L)	219.3 ± 15.38	217.3 ± 13.40	207.7 ± 11.3	206.6 ± 10.90

Values are expressed as mean± SEM. * *p* < 0.05, 3rd-month values of the resveratrol group compared to the baseline values. # *p* < 0.05, resveratrol vs. placebo group. Baseline: measured values at randomization in the resveratrol or in the placebo group; 3rd month: patients treated with resveratrol or placebo for 3 months. ALT: alanine aminotransferase, AST: aspartate aminotransferase, BUN: blood urea nitrogen, CRP: C-reactive protein, cTroponin T: cardiac Troponin T, Hct: hematocrit, HDL-C: high-density lipoprotein, Hgb_A1c_: hemoglobin A1c, LDL-C: low-density lipoprotein, PLT: platelet count, Se Fe: serum iron level, TC: total cholesterol, TG: triglyceride, Transf. sat.: transferrin saturation and WBC: white blood cell count.

**Table 5:** BMI, general hemodynamic and ECG parameters.

Parameters	Baseline		3rd Month	
	Resveratrol	Placebo	Resveratrol	Placebo
BW (kg)	85.53 ± 3.09	89.97 ± 5.06	84.77 ± 3.19	90.80 ± 4.77
BMI (kg/m^2^)	29.31 ± 0.99	31.04 ± 1.46	29.06 ± 1.01	31.34 ± 1.39
Systolic BP (Hgmm)	132.5 ± 3.41	128.8 ± 3.80	131.6 ± 3.21	125.8 ± 2.65
Diastolic BP (Hgmm)	79.10 ± 2.33	80.13 ± 2.66	77.83 ± 1.82	76.30 ± 2.24
HR (beat/min)	72.20 ± 2.75	76.93 ± 2.50	70.04 ± 2.87	70.73 ± 2.26
PQ (ms)	164.1 ± 5.37	187.9 ± 5.67	162.1 ± 6.28	189.9 ± 5.68
QRS (ms)	127.8 ± 5.35	137.3 ± 6.19	136.5 ± 5.68	132.9 ± 5.84
QTc (ms)	463.7 ± 5.03	476.9 ± 6.65	469.9 ± 5.99	471.2 ± 5.29

Values are expressed as mean ± SEM. Baseline: measured values at randomization in the resveratrol or in the placebo group, 3rd month: patients treated with resveratrol or placebo for 3 months, BMI: body mass index, BP: blood pressure, BW: body weight, HR: heart rate, PQ: PQ interval on an electrocardiogram (ECG), QRS: the wide of the QRS complex on the ECG and QTc: heart rate-corrected QT interval on ECG.

**Table 6:** Left ventricular echocardiographic parameters.

Echocardiographic Parameters	Baseline		3rd Month	
	Resveratrol	Placebo	Resveratrol	Placebo
EF (Quinone, %)	29.19 ± 1.04	30.16 ± 1.10	33.40 ± 1.20 ** ^#^	29.79 ± 0.95
EF (Simson, %)	30.06 ± 1.04	31.70 ± 1.27	34.60 ± 1.38 ** ^#^	30.41 ± 1.36
LVIDd (mm)	65.5 ± 1.55	62.83 ± 1.12	65.09 ± 1.44	62.62 ± 1.12
LVIDs (mm)	54.87 ± 1.67	51.90 ± 1.06	52.88 ± 1.62	51.67 ± 1.06
LVEDV (mL)	182.4 ± 11.73	157.3 ± 7.92	176.7 ± 10.05	160.1 ± 8.01
LVESV (mL)	129.0 ± 9.37	106.7 ± 5.80	116.3 ± 8.00 **	111.4 ± 5.60
SV (mL)	52.85 ± 2.82	50.69 ± 3.38	60.46 ± 3.13 ** ^#^	48.56 ± 3.84
SW (mm)	11.47 ± 0.28	11.83 ± 0.31	11.43 ± 0.29	11.83 ± 0.31
PW (mm)	11.03 ± 0.17	11.17 ± 0.20	11.20 ± 0.17	11.14 ± 0.21
GLS (%)	−8.40 ± 0.62	−8.80 ± 0.75	−9.58 ± 0.73 * ^#^	−8.45 ± 0.75
E (m/s)	0.89 ± 0.04	0.98 ± 0.05	0.80 ± 0.05 * ^#^	1.00 ± 0.05
A (m/s)	0.78 ± 0.04	0.71 ± 0.05	0.78 ± 0.04	0.76 ± 0.06
E’	0.05 ± 0.002	0.05 ± 0.003	0.06 ± 0.003	0.05 ± 0.003
E/A ratio	1.26 ± 0.12	1.57 ± 0.20	1.18 ± 0.15 ^#^	1.54 ± 0.19
E/E’ ratio	17.65 ± 1.31	19.32 ± 1.21	15.55 ± 1.43 ^#^	19.94 ± 1.43

Values are expressed as mean ± SEM. * *p* < 0.05, 3rd month values of the resveratrol group compared to the baseline values. ** *p* < 0.01, 3rd month values of the resveratrol group compared to the baseline values. # *p* < 0.05, resveratrol vs. placebo group at the 3rd month. A: late diastolic ventricular filling velocity, A’: late diastolic mitral annular velocity, GLS: global longitudinal strain of the left ventricle, E: early diastolic ventricular filling velocity, E’: early diastolic mitral annular velocity, EF: ejection fraction, LVEDV: left ventricular end-diastolic volume, LVESV: left ventricular end-systolic volume, LVIDd: diastolic left ventricular inner diameter, LVIDs: systolic left ventricular inner diameter, PW: posterior wall thickness, SV: stroke volume and SW: septal wall thickness.

**Table 7:** Echocardiographic parameters of the right ventricle, right atrium and left atrium.

Echocardiographic Parameters	Baseline		3rd Month	
	Resveratrol	Placebo	Resveratrol	Placebo
RVIDd-mid (mm)	27.86 ± 1.14	23.90 ± 1.02	25.09 ± 1.09	26.96 ± 0.98 *
RVIDd-basal (mm)	37.43 ± 1.43	34.14 ± 1.10	34.83 ± 1.12 *	37.03 ± 1.17 *
RVIDs (mm)	30.34 ± 1.21	29.43 ± 1.26	28.00 ± 1.05 *	29.86 ± 1.13
TAPSE (mm)	16.24 ± 0.74	16.58 ± 0.74	17.96 ± 0.82 ^#^	15.76 ± 0.63
LA long axis (mm)	64.03 ± 1.68	64.21 ± 1.25	61.79 ± 1.72 * ^#^	64.17 ± 1.14
LA short axis (mm)	46.14 ± 0.98	45.38 ± 0.93	45.29 ± 1.03	45.45 ± 0.99
LA area (cm^2^)	27.11 ± 1.33	27.95 ± 1.10	25.99 ± 1.35	28.05 ± 1.10
LAEDV (mL)	59.96 ± 5.24	63.68 ± 4.21	50.74 ± 3.14 ^#^	66.24 ± 4.79
LAESV (mL)	87.52 ± 5.37	87.95 ± 4.69	78.12 ± 3.99 * ^#^	88.00 ± 4.92
RA long axis (mm)	58.34 ± 2.13	58.59 ± 1.63	56.72 ± 2.28	60.10 ± 1.65
RA short axis (mm)	41.34 ± 1.21	41.00 ± 1.11	39.86 ± 1.37	40.31 ± 1.04
RA area (cm^2^)	21.20 ± 2.12	21.28 ± 1.30	20.08 ± 2.09	21.56 ± 1.33
RAEDV (mL)	42.87 ± 4.44	44.24 ± 5.18	34.50 ± 3.47 * ^#^	43.14 ± 4.68
RAESV (mL)	54.63 ± 5.15	60.23 ± 5.53	49.32 ± 3.50 ^#^	63.04 ± 5.64
IVC (mm)	16.08 ± 1.01	17.38 ± 0.97	15.24 ± 0.89 ^#^	18.79 ± 0.84

Values are expressed as mean ± SEM. * *p* < 0.05, 3rd month values of the resveratrol or placebo group compared to the baseline values. # *p* < 0.05, resveratrol vs. placebo group at the 3rd month. Baseline: measured values at randomization in the resveratrol or in the placebo group; 3rd month: patients treated with resveratrol or placebo for 3 months. IVC: inferior vena cava, LA: left atrium, LAEDV: left atrium end-diastolic volume, LAESV: left atrium end-diastolic volume, RA: right atrium; RAEDV: right atrium end-diastolic volume, RAESV: right atrium end-diastolic volume, RVIDd-basal: diastolic basal right ventricular inner diameter, RVIDd-mid: diastolic mid-right ventricular inner diameter, RVIDs: systolic right ventricular inner diameter and TAPSE: tricuspid annular plane systolic excursion.

**Table 8:** The effect of resveratrol on the spirometric parameters.

Spirometric Parameters	Baseline		3rd Month	
	Resveratrol	Placebo	Resveratrol	Placebo
FVC (%)	75.38 ± 3.31	74.41 ± 3.75	79.27 ± 3.07 *	76.91 ± 4.08
FEV_1_ (%)	64.67 ± 3.22	65.10 ± 3.67	67.38 ± 3.00	65.90 ± 3.98
FEV_1_/FVC ratio (%)	89.79 ± 1.84	94.63 ± 2.18	89.29 ± 2.33	93.74 ± 2.80
PEF (%)	56.50 ± 3.01	55.91 ± 3.67	57.13 ± 3.28	59.09 ± 3.89
FEF 25-75 (%)	55.92 ± 3.79	55.50 ± 3.80	55.81 ± 3.98	58.38 ± 4.82
MEF75 (%)	58.50 ± 3.60	56.68 ± 3.50	60.85 ± 3.99	60.90 ± 4.33
MEF50 (%)	55.65 ± 4.10	52.95 ± 3.83	55.35 ± 3.77	57.14 ± 4.09
MEF25 (%)	52.35 ± 4.30	55.95 ± 5.16	53.23 ± 4.73	56.21 ± 4.94
IVC (%)	83.35 ± 3.65	81.32 ± 3.60	90.27 ± 3.39 *	86.36 ± 3.91

Values are expressed as mean± SEM. * *p* < 0.05, 3rd month values of resveratrol group compared to the baseline values. Baseline: measured values at randomization in resveratrol or in placebo group; 3rd month: patients treated with resveratrol or placebo for 3 months. FEF 25–75%: forced expiratory flow 25–75%, FEV_1_: forced expiratory volume, FVC: forced vital capacity, IVC: inspiratory vital capacity, MEF: maximal expiratory flows at 25%, 50% or 75% of FVC, PEF: peak expiratory flow.

## References

[1] Ponikowski P., Voors A.A., Anker S.D., Bueno H., Cleland J.G.F., Coats A.J.S., Falk V., Gonzalez-Juanatey J.R., Harjola V., Jankowska E.A. (2016). ESC Guidelines for the diagnosis and treatment of acute and chronic heart failure. The Task Force for the diagnosis and treatment of acute and chronic heart failure of the European Society of Cardiology (ESC). Eur. Heart J..

[2] Greenberg B.H. (2016). A Treatment Approach for Patients with Chronic Systolic Heart Failure. Rev. Cardiovasc. Med..

[3] Mosterd A., Hoes A.W. (2007). Clinical epidemiology of heart failure. Heart.

[4] Ziaeian B., Fonarow G.C. (2016). Epidemiology and aetiology of heart failure. Nat. Rev. Cardiol..

[5] Swedberg K., Komajda M., Böhm M., Borer J.S., Ford I., Dubost-Brama A., Lerebours G., Tavazzi L. (2010). Ivabradine and outcomes in chronic heart failure (SHIFT): A randomised placebo-controlled study. Lancet.

[6] McMurray J.J., Packer M., Desai A.S., Gong J., Lefkowitz M.P., Rizkala A.R., Rouleau J.L., Shi V.C., Solomon S.D., Swedberg K. (2014). PARADIGM-HF Investigators and Committees. Angiotensin-neprilysin inhibition versus enalapril in heart failure. N. Engl. J. Med..

[7] Sauer A.J., Cole R., Jensen B.C., Pal J., Sharma N., Yehya A., Vader J. (2019). Practical guidance on the use of sacubitril/valsartan for heart failure. Heart Fail. Rev..

[8] Bui A.L., Horwich T.B., Fonarow G.C. (2011). Epidemiology and risk profile of heart failure. Nat. Rev. Cardiol..

[9] Kim D.H., Chien F.J., Eisen H.J. (2017). Pharmacologic Management for Heart Failure and Emerging Therapies. Curr. Cardiol. Rep..

[10] Patel C., Deoghare S. (2015). Heart failure: Novel therapeutic approaches. J. Postgrad. Med..

[11] Tsutsui H., Kinugawa S., Matsushimam S. (2011). Oxidative stress and heart failure. Am. J. Physiol. Heart Circ. Physiol..

[12] Oyewole A.O., Birch-Machin M.A. (2015). Mitochondria-targeted antioxidants. FASEB J..

[13] Raj P., Lieben Louis X., Thandapilly S.J., Movahed A., Zieroth S., Netticadan T. (2014). Potential of resveratrol in the treatment of heart failure. Life Sci..

[14] Gupta P.K., DiPette D.J., Supowit S.C. (2014). Protective effect of resveratrol against pressure overload-induced heart failure. Food Sci. Nutr..

[15] Wojciechowski P., Juric D., Louis X.L., Thandapilly S.J., Yu L., Taylor C., Netticadan T. (2010). Resveratrol arrests and regresses the development of pressure over load-but not volume overload-induced cardiac hypertrophy in rats. J. Nutr..

[16] Danz E.D., Skramsted J., Henry N., Bennett J.A., Keller R.S. (2009). Resveratrol prevents doxorubicin cardiotoxicity through mitochondrial stabilization and the Sirt1pathway. Free Radic. Biol. Med..

[17] Xia N., Daiber A., Förstermann U., Li H. (2017). Antioxidant effects of resveratrol in the cardiovascular system. Br. J. Pharmacol..

[18] Lagouge M., Argmann C., Gerhart-Hines Z., Meziane H., Lerin C., Daussin F., Messadeq N., Milne J., Lambert P., Elliott P. (2006). Resveratrol improves mitochondrial function and protects against metabolic disease by activating SIRT1 and PGC-1alpha. Cell.

[19] Gu X.S., Wang Z.B., Ye Z., Lei J.P., Li L., Su D.F., Zheng X. (2014). Resveratrol, an activator of SIRT1, upregulates AMPK and improves cardiac function in heart failure. Genet. Mol. Res..

[20] Dyck G.J.B., Raj P., Zieroth S., Dyck J.R.B., Ezekowitz J.A. (2019). The Effects of Resveratrol in Patients with Cardiovascular Disease and Heart Failure: A Narrative Review. Int. J. Mol. Sci..

[21] Xia N., Forstermann U., Li H. (2017). Effects of resveratrol on eNOS in the endothelium and the perivascular adipose tissue. Ann. N. Y. Acad. Sci..

[22] Schwager J., Richard N., Widmer F., Raederstorff D. (2017). Resveratrol distinctively modulates the inflammatory profiles of immune and endothelial cells. BMC Complement. Altern. Med..

[23] Carrizzo A., Puca A., Damato A., Marino M., Franco E., Pompeo F., Traficante A., Civitillo F., Santini L., Trimarco V. (2013). Resveratrol improves vascular function in patients with hypertension and dyslipidemia by modulating NO metabolism. Hypertension.

[24] Simental-Mendía L.E., Guerrero-Romero F. (2019). Effect of resveratrol supplementation on lipid profile in subjects with dyslipidemia: A randomized double-blind, placebo-controlled trial. Nutrition.

[25] Riba A., Deres L., Sumegi B., Toth K., Szabados E., Halmosi R. (2017). Cardioprotective Effect of Resveratrol in a Postinfarction Heart Failure Model. Oxid. Med. Cell Longev..

[26] Sung M.M., Dyck J.R.B. (2015). Therapeutic potential of resveratrol in heart failure. Ann. N. Y. Acad. Sci..

[27] Williams B., Mancia G., Spiering W., Rosei E.A., Azizi M., Burnier M., Clement D.L., Coca A., de Simone G., Dominiczak A. (2018). 2018 ESC/ESH Guidelines for the management of arterial hypertension The Task Force for the management of arterial hypertension of the European Society of Cardiology (ESC) and the European Society of Hypertension (ESH). Eur. Heart J..

[28] Crapo R.O., Casaburi R., Coates A.L. (2002). ATS statement: Guidelines for the six-minute walk test. Am. J. Respir. Crit. Care Med..

[29] Johnson B.D., Beck K.C., Olson L.J., O’Malley K.A., Allison T.G., Squires R.W., Gau G.T. (2000). Ventilatory constraints during exercise in patients with chronic heart failure. Chest.

[30] Dyer M.T.D., Goldsmith K.A., Sharples L.S., Buxton M.J. (2010). A review of health utilities using the EQ-5D in studies of cardiovascular disease. Health Qual. Life Outcomes.

[31] Karlsson J., Emgard M., Brundin P., Burkitt M.J. (2000). Trans-Resveratrol protects embryonic mesencephalic cells from tertButyl hydroperoxide: Electron paramagnetic resonance spin trapping evidence for a radical scavenging mechanism. J. Neurochem..

[32] Spanier G., Xu H., Xia N., Tobias S., Deng S., Wojnowski L., Forstermann U., Li H. (2009). Resveratrol reduces endothelial oxidative stress by modulating the gene expression of superoxide dismutase 1 (SOD1), glutathione peroxidase 1 (GPX1) and NADPH oxidase subunit (NOX4). J. Physiol. Pharmacol..

[33] Thirunavukkarasu M., Penumathsa S.V., Koneru S., Juhasz B., Zhan L., Otani H., Bagchi D., Das D.K., Maulik N. (2007). Resveratrol alleviates cardiac dysfunction in streptozotocin-induced diabetes: Role of nitric oxide, thioredoxin and heme oxygenase. Free Radic. Biol. Med..

[34] Yildiz F., Terzi A., Coban S., Celik H., Aksoy N., Bitiren M., Cakir H., Ozdogan M.K. (2009). Protective effects of resveratrol on small intestines against intestinal ischemia-reperfusion injury in rats. J. Gastroenterol. Hepatol..

[35] Sharma S., Chopra K., Kulkarni S.K., Agrewala J.N. (2007). Resveratrol and curcumin suppress immune response through CD28/CTLA-4 and CD80 co-stimulatory pathway. Clin. Exp. Immunol..

[36] Shen M.Y., Hsiao G., Liu C.L., Fong T.H., Lin K.H., Chou D.S., Sheu J.R. (2007). Inhibitory mechanisms of resveratrol in platelet activation: Pivotal roles of p38 MAPK and NO/cyclic GMP. Br. J. Haematol..

[37] Cho I.J., Ahn J.Y., Kim S., Choi M.S., Ha T.Y. (2008). Resveratrol attenuates the expression of HMG-CoA reductase mRNA in hamsters. Biochem. Biophys. Res. Commun..

[38] Baur J.A., Pearson K.J., Price N.L., Jamieson H.A., Lerin C., Kalra A., Prabhu V.V., Allard J.S., Lopez-Lluch G., Lewis K. (2006). Resveratrol improves health and survival of mice on a high-calorie diet. Nature.

[39] Lin Y., Zhu M., Su Z. (2015). The pursuit of balance: An overview of covariate-adaptive randomization techniques in clinical trials. Contemp. Clin. Trials.

[40] Wilson D.J., North N., Wilson R.A. (1998). Comparison of Left Ventricular Ejection Fraction Calculation Methods. Echocardiography.

[41] Cameli M., Mondillo S., Righini F.M., Lisi M., Dokollari A., Lindqvist P., Maccherini M., Henein M. (2016). Left Ventricular Deformation and Myocardial Fibrosis in Patients with Advanced Heart Failure Requiring Transplantation. J. Card. Fail..

[42] Qin F., Siwik D.A., Luptak I., Hou X., Wang L., Higuchi A., Weisbrod R.M., Ouchi N., Tu V.H., Calamaras T.D. (2012). The polyphenols resveratrol and S17834 prevent the structural and functional sequelae of diet-induced metabolic heart disease in mice. Circulation.

[43] Sulaiman M., Matta M.J., Sunderesan N.R., Gupta M.P., Periasamy M., Gupta M. (2010). Resveratrol, an activator of SIRT1, upregulates sarcoplasmic calcium ATPase and improves cardiac function in diabetic cardiomyopathy. Am. J. Physiol. Heart Circ. Physiol..

[44] Matsumura N., Takahara S., Maayah Z.H., Parajuli N., Byrne N.J., Shoieb S.M., Soltys C.M., Beker D.L., Masson G., El-Kadi A.O.S. (2018). Resveratrol improves cardiac function and exercise performance in MI-induced heart failure through the inhibition of cardiotoxic HETE metabolites. J. Mol. Cell Cardiol..

[45] Magyar K., Halmosi R., Palfi A., Feher G., Czop L., Fulop A., Battyany I., Sumegi B., Toth K., Szabados E. (2012). Cardioprotection by resveratrol: A human clinical trial in patients with stable coronary artery disease. Clin. Hemorheol. Microcirc..

[46] Hart N., Sarga L., Csende Z., Koltai E., Koch L.G., Britton S.L., Davies K.J., Kouretas D., Wessner B., Radak Z. (2018). Resveratrol enhances exercise training responses in rats selectively bred for high running performance. Food Chem. Toxicol..

[47] Voduc N., la Porte C., Tessier C., Mallick R., Cameron D.W. (2014). Effect of resveratrol on exercise capacity: A randomized placebo-controlled crossover pilot study. Appl. Physiol. Nutr. Metab..

[48] Gliemann L., Schmidt J.F., Olesen J., Biensø R.S., Peronard S.L., Grandjean S.U., Mortensen S.P., Nyberg M., Bangsbo J., Pilegaard H. (2013). Resveratrol blunts the positive effects of exercise training on cardiovascular health in aged men. J. Physiol..

[49] Amin H.Z., Amin L.Z., Wijaya I.P. (2017). Galectin-3: A novel biomarker for the prognosis of heart failure. Clujul. Med..

[50] Brinchmann M.F., Patel D.M., Iversen M.H. (2018). The Role of Galectins as Modulators of Metabolism and Inflammation. Mediators Inflamm..

[51] Militaru C., Donoiu I., Craciun A., Scorei I.D., Bulearca A.M., Scorei R.I. (2013). Oral resveratrol and calcium fructoborate supplementation in subjects with stable angina pectoris: Effects on lipid profiles, inflammation markers, and quality of life. Nutrition.

[52] Gaggin H.K., Januzzi J.L. (2013). Biomarkers and diagnostics in heart failure. Biochim. Biophys. Acta.

[53] Chase S.C., Fermoyle C.C., Wheatley C.M., Schaefer J.J., Olson L.J., Johnson B.D. (2018). The effect of diuresis on extravascular lung water and pulmonary function in acute decompensated heart failure. ESC Heart Fail..

[54] Gehlbach B.K., Geppert E. (2004). The pulmonary manifestations of left heart failure. Chest.

[55] Malaguarnera L. (2019). Influence of resveratrol on the immune response. Nutrients.

[56] Yan A.T., Yan R.T., Cushman M., Redheuil A., Tracy R.P., Arnett D.K., Rosen B.D., McClelland R.L., Bluemke D.A., Lima J.A.C. (2010). Relationship of interleukin-6 with regional and global left-ventricular function in asymptomatic individuals without clinical cardiovascular disease: Insights from the Multi-Ethnic Study of Atherosclerosis. Eur. Heart J..

[57] Mann D.L. (2015). Innate immunity and the failing heart: The cytokine hypothesis revisited. Circ. Res..

